# Statistical quality indicators for electron-density maps

**DOI:** 10.1107/S0907444911035918

**Published:** 2012-03-16

**Authors:** Ian J. Tickle

**Affiliations:** a Astex Pharmaceuticals, 436 Science Park, Milton Road, Cambridge CB4 0QA, England

**Keywords:** difference density, electron density, model accuracy, model precision, real-space *R*, real-space correlation coefficient, real-space difference density *Z* score, real-space observed density *Z* score, structure validation

## Abstract

The commonly used validation metrics for the local agreement of a structure model with the observed electron density, namely the real-space *R* (RSR) and the real-space correlation coefficient (RSCC), are reviewed. It is argued that the primary goal of all validation techniques is to verify the accuracy of the model, since precision is an inherent property of the crystal and the data. It is demonstrated that the principal weakness of both of the above metrics is their inability to distinguish the accuracy of the model from its precision. Furthermore, neither of these metrics in their usual implementation indicate the statistical significance of the result. The statistical properties of electron-density maps are reviewed and an improved alternative likelihood-based metric is suggested. This leads naturally to a χ^2^ significance test of the difference density using the real-space difference density *Z* score (RSZD). This is a metric purely of the local model accuracy, as required for effective model validation and structure optimization by practising crystallographers prior to submission of a structure model to the PDB. A new real-space observed density *Z* score (RSZO) is also proposed; this is a metric purely of the model precision, as a substitute for other precision metrics such as the *B* factor.

## Abstract 2

A likelihood-based metric for scoring the local agreement of a structure model with the observed electron density is described.

## 1. Background

Global metrics of accuracy of the structure model (such as *R*_free_) do not identify local errors in a model. A better metric of local accuracy of the model is consistency with the electron density in real space. This assumes that the electron density itself, and therefore the phases from which it is derived, are accurate. This is a reasonable assumption because density-based validation is normally performed near the completion of refinement when the model is mostly correct and only a small number of minor errors remain to be resolved.

## 2. Outline

### 2.1. Review existing real-space electron-density metrics

(i) Real-space *R* (RSR).
(ii) Real-space correlation coefficient (RSCC).
(iii) Why *both* these metrics are sub-optimal as validation metrics.
(iv) What are the characteristics of an optimal metric?

### 2.2. Other issues related to current implementations of RSR and RSCC

The sensitivity of any real-space metric of electron density depends critically on the following.

(i) Accurate representation of the electron density corresponding to the model (ρ_calc_).
(ii) Accurate scaling of the model density *ρ*_calc_ to the observed density ρ_obs_.
(iii) Accurate estimation of the limiting radius of the metric in the density: this requires an accurate calculation of the atomic peak density profile as actually observed in the map.

### 2.3. Proposed new electron-density metrics based on the difference Fourier map

(i) The *Q*–*Q* difference plot.
(ii) The real-space difference density *Z* score (RSZD).
(iii) The real-space observed density *Z* score (RSZO).

### 2.4. Other issues related to calculation of electron-density maps

(i) Correct treatment of centric reflections in map calculations.
(ii) Correct treatment of overlapping atom densities.
(iii) Testing of statistical significance of a real-space metric.

## 3. Definitions

### 3.1. Accuracy *versus* precision

**Figure 1:**
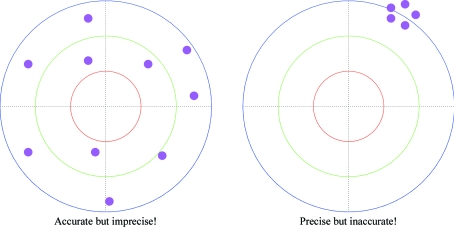
Simple illustration of the difference between accuracy and precision.

Accuracy means ‘how close are the results on average to the truth (regardless of their precision)?’ (see Fig. 1 ▶ for a simple illustration). Hence, accuracy is measured by observed error (or often just ‘error’). Provided the experimental data are accurate, accuracy is a property of the model: it can be improved by model building and refinement using the current data.

Precision means ‘if you were to repeat the experiment, how much would you expect the results to vary (regardless of their accuracy)?’. Hence, precision is measured by expected error (usually known as ‘uncertainty’). Provided the refinement is performed optimally, model precision is an inherent property of the crystal and the experimental data: it can only be improved by making a more ordered crystal form and/or by collecting better (*e.g.* more accurate and/or higher resolution) data.

### 3.2. What do we actually mean by ‘validation’?

In usage, the term ‘validation’ appears to have the following two quite distinct meanings.

(i) Validation of the structure model: is it the model that is most consistent with the data (diffraction experiment + prior information)? Assuming the data are accurate, the model that is most consistent with the data (*i.e.* that corresponding to the global maximum of the total likelihood, assuming minimal overfitting to errors in the data) is the most *accurate* one: this is what crystallographers usually mean by ‘validation’.
(ii) Validation of the *utility* of the model: how useful is the model in terms of the reliability of the conclusions (*e.g.* about structure–function relationships) that you or others wish to draw from it, assuming that the accuracy of the model has been verified? Now the optimal measure of ‘reliability’ is the *precision* of the model: this is likely to be what end-users of structures understand by validation.

### 3.3. What is the goal of validation?

Ideally, if the goal of validation is to measure accuracy [meaning (i)], then for maximum sensitivity the validation metric should correlate only with model accuracy. Similarly, if the goal is to measure precision [meaning (ii)], then the metric should correlate only with model precision. Otherwise, it is impossible to tell how much of the observed effect on the validation metric to ascribe to lack of accuracy and how much to ascribe to lack of precision.

## 4. Current methods for validation in real space using the electron density

(i) Real-space *R* (version 1; Jones *et al.*, 1991 ▶), implemented in *O* (Uppsala Software Factory).
(ii) Real-space *R* (version 2; Gerard Kleywegt), implemented in *MAPMAN* (USF).
(iii) Real-space *R* (version 3; Eleanor Dodson), implemented in *SFALL* + *OVERLAPMAP* (*CCP*4; Winn *et al.*, 2011 ▶).
(iv) Real-space correlation coefficient (RSCC) in *O*, *MAPMAN* and *SFALL* + *OVERLAPMAP*.

### 4.1. Real-space *R* (RSR; version of Jones and coworkers)

The real-space *R* (version of Jones and coworkers) is computed for a group of atoms (*e.g.* main-chain or side-chain atoms in a single residue). The observed and calculated electron densities are sampled on a grid which covers the atoms. For ρ_calc_ a single Gaussian atom density model with fixed overall *B* factor is used. This estimate of ρ_calc_ is not on an absolute scale so must be rescaled with a single overall scale factor to ρ_obs_. The real-space *R* is then defined as 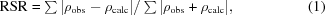
where the sum is over grid points within a specified limiting radius centred on each atom. The range of RSR is 0 (‘good’) to ∼1 (‘bad’). Note that ρ_obs_ and ρ_calc_ may be zero or negative owing to omission of the *F*_000_ term, incomplete data or limited resolution (‘series termination’).

#### 4.1.1. Issues specific to the RSR version of Jones and co­workers

**Figure 2:**
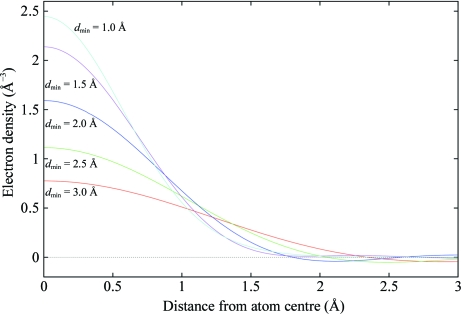
Theoretical electron-density function plotted for an O atom (*B* = 20 Å^2^) showing the dependence of the atom density profile on the resolution cutoff *d*_min_.

The RSR version of Jones and coworkers assumes a fixed peak profile for all atoms: in reality, it will depend on scattering factor (atom type), *B* factor, data completeness and maximum and minimum *d*-spacings (resolution limits). Even if the atomic scattering factor is assumed to be Gaussian, the resolution-limited electron-density profile is the convolution of that three-dimensional Gaussian with a sphere enclosing constant scattering power and zero scattering outside the sphere (Blundell & Johnson, 1976 ▶, §5.4). The truncated Fourier transform of the atomic scattering factor *f*(*s*) between sin(θ)/λ limits *s*_min_ and *s*_max_, assuming an isotropic *B* factor, gives
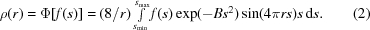
Fig. 2 ▶ shows this function plotted for an O atom (*s*_min_ = 0 and *B* = 20 Å^2^), showing the dependence of the atom density profile on the resolution cutoff *d*_min_ (= 0.5/*s*_max_). The integral (2) is computed numerically using Legendre–Gauss quadrature: *f*(*s*) is a sum of four Gaussians fitted to tabulated atomic scattering factors (*International Tables for Crystallography,* 1999 ▶; the parameters of the Gaussians for a given element were taken from the *CCP*4-installed library file $CLIBD/atomsf.lib).

### 4.2. Real-space *R* (versions of Kleywegt and Dodson)

**Figure 3:**
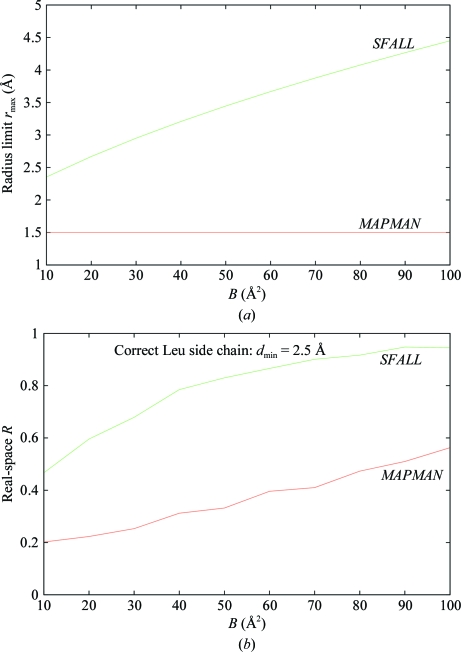
(*a*) Plot of the atomic radius limits used in the distributed versions of *MAPMAN* and *SFALL* as a function of the atomic *B* factor, showing the large discrepancy in the values used; (*b*) plot of the real-space *R* for a Leu side chain with simulated normally distributed random errors in the electron density (resolution cutoff *d*_min_ = 2.5 Å) based on the atomic radius limits shown in (*a*). This shows the effect on the RSR of the large difference in radius limits and also the dependence of the RSR on the *B* factor in each case.

The real-space *R* versions of Kleywegt and Dodson are defined as for the Jones version, except that ρ_calc_ obtained by a Fourier transform of the calculated structure factors is used instead of Gaussian atomic peak profiles and hence all factors that affect the atomic density profiles are automatically taken into account. The values of the limiting radii used are chosen arbitrarily and vary between implementations (Fig. 3 ▶ *a*); this causes RSR to vary wildly according to the software used (Fig. 3 ▶ *b*). The values may be fixed (*e.g. r*_max_ = 1.5 Å in *MAPMAN*) or may depend only on *B* factor [*e.g. r*_max_ = 2.5(*B* + 25)^1/2^/2π Å in *SFALL*].

**Figure 4:**
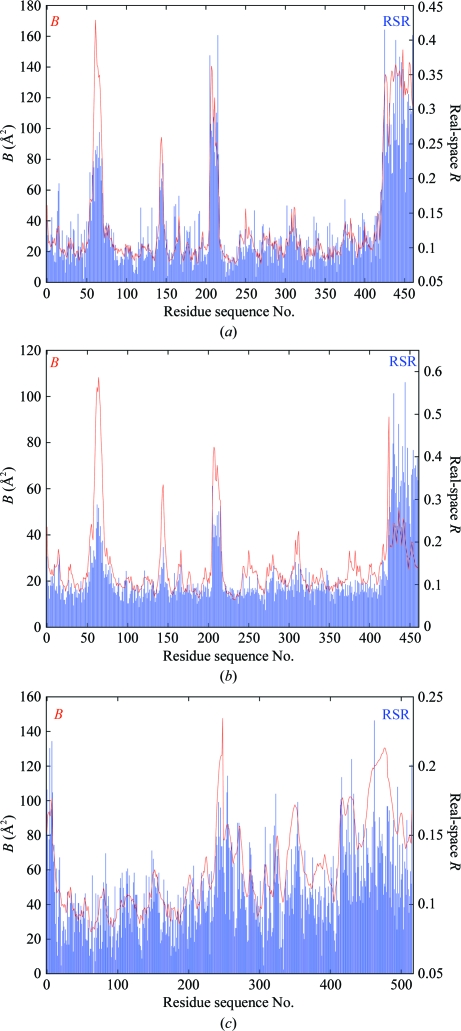
(*a*) Plot of average *B* factor and real-space *R* as a function of residue sequence number for the main-chain atoms (including C^β^) of the 1f83 structure based on the atomic radius limits defined in §[Sec sec5.6]5.6; (*b*) the same for 3g94; (*c*) the same for 2w96. The strong correlation of RSR with *B* factor is evident and is clearly not related to inaccuracies in the model.

Fig. 4 ▶ shows plots of the main-chain mean *B* factor and RSR *versus* residue sequence number for PDB entries 1f83 and 3g94 (both for botulinum neurotoxin type B catalytic domain in complex with synaptobrevin II; Hanson & Stevens, 2000 ▶) and 2w96 (cyclin-dependent kinase 4 complex with cyclin D1; Day *et al.*, 2009 ▶). Entry 1f83 was found to contain gross in­accuracies: the errors were subsequently corrected and 1f83 was obsoleted (2007) and replaced by 3g94; the latter was then also retracted (Hanson & Stevens, 2009 ▶) because the im­precise density observed for the ligand did not support the conclusions drawn. The CDK4–cyclin D1 complex was determined concurrently and independently to that of Day *et al.* (2009 ▶) by Takaki *et al.* (2009 ▶) and proved to be identical within the expected limits of precision. These three structures thus provide a nice comparative test of the various real-space density scores: we can take 1f83 and 3g94 as representatives of an inaccurate and an imprecise structure, respectively.

### 4.3. Real-space correlation coefficient (RSCC)

**Figure 5:**
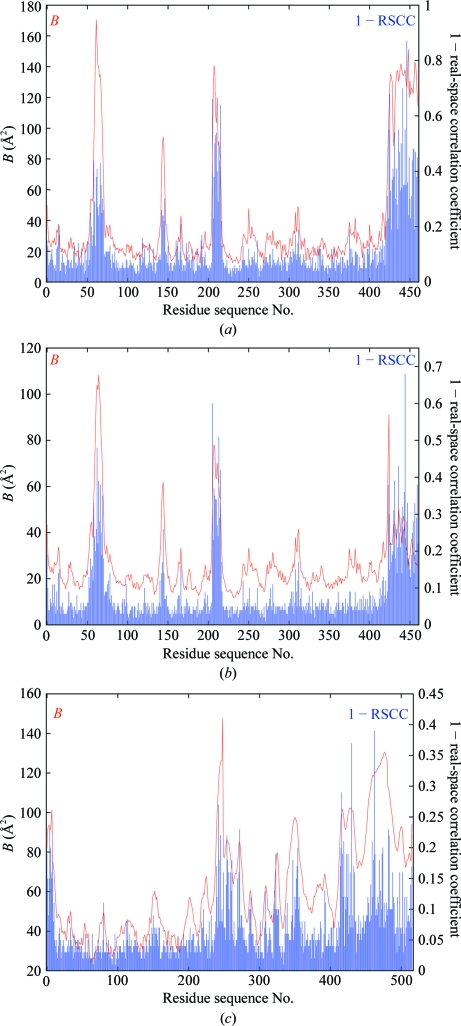
(*a*) Plot of average *B* factor and (1 − real-space sample correlation coefficient) as a function of residue sequence number for the main-chain atoms (including C^β^) of the 1f83 structure based on the atomic radius limits defined in §[Sec sec5.6]5.6; (*b*) the same for 3g94; (*c*) the same for 2w96. The strong correlation of RSCC with *B* factor is evident and is clearly not related to inaccuracies in the model.

RSCC is the standard linear sample correlation coefficient (also known as ‘Pearson’s product–moment sample correlation coefficient’),
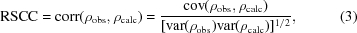
where var(·’;) is the *sample* variance and cov(·) is the *sample* covariance (*i.e.* relative to the *sample* means). The values of the limiting radii are as for RSR and the range of RSCC is from ∼0 (‘bad’) to 1 (‘good’). Fig. 5 ▶ shows plots of the main-chain mean *B* factor and RSCC *versus* residue sequence number for PDB entries 1f83, 3g94 and 2w96; the ordinate plotted is (1 − RSCC) for easier comparison with the RSR and *B*-factor plots.

**Figure 6:**
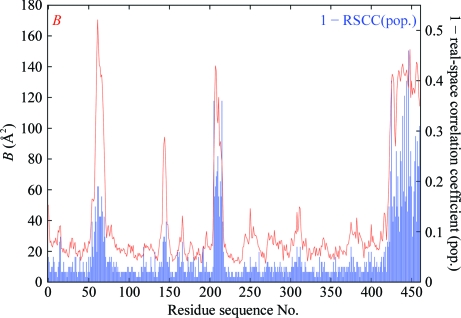
Plot of average *B* factor and (1 − real-space ‘population’ correlation coefficient) as a function of residue sequence number for the main-chain atoms (including C^β^) of the 1f83 structure based on the atomic radius limits defined in §[Sec sec5.6]5.6. This shows the same strong correlation with *B* factor as the standard ‘sample’ RSCC, but the advantage is that it detects weaker correlations (note the difference in the scale for 1 − RSCC).

Note that the alternative ‘*population*’ correlation coefficient, which measures correlations of the deviations in ρ_obs_ and ρ_calc_ from the overall population means (*i.e.* zero) instead of correlations of deviations from the local sample means, is more sensitive to lower correlations than the sample CC (Fig. 6 ▶).

### 4.4. Issues for all versions of RSR and RSCC

#### 4.4.1. Limiting atom radius

Real-space metrics are likely to depend critically on the value of the limiting atom radius used. For RSR and RSCC the peak profile is assumed to either be fixed or to be a function of *B* factor only, whereas in reality the peak profile and therefore the optimal limiting radius also depends on scattering factors (atom type) and maximum and minimum *d*-spacings (resolution limits). If the radius is too small, insufficient density is included and the ‘signal’ component is reduced; if it is too large, the ‘noise’ increases. Either way, the signal-to-noise ratio deteriorates.

#### 4.4.2. Scaling of density

Inappropriate scaling of the ρ_calc_ density will inevitably introduce errors in the calculation of the various metrics. In some implementations the ‘un­weighted’ *F*_c_ is used and ρ_calc_ must be rescaled to ρ_obs_ using a single overall scale factor. The scale factor of *F*_c_ to the Fourier coefficient (2*mF*_o_ − *DF*_c_) is resolution-dependent so a single scale factor is not appropriate. The required resolution-dependent scale factor is in fact already calculated by the refinement program: *D*. Hence, the use of *F*_c_ with a single resolution-independent scale factor is likely to introduce errors; the already correctly scaled coefficient for ρ_calc_ is *DF*_c_. Note that the use of RSCC implicitly assumes that a single overall scale factor is appropriate.

### 4.5. Other issues for all versions of RSR and RSCC

Most implementations of RSR and RSCC ignore overlaps in contributions to ρ_obs_ from adjacent groups, so that atoms at the boundaries between different groups contribute twice. Also, the testing of statistical significance (*i.e.* how meaningful are the calculated values of the validation metric?) is not possible with RSR as defined (using absolute values), since this form of *R* is not found in any published statistical tables. Significance testing of RSCC is in principle possible, although to the author’s knowledge this has never been used in practice.

The major issue with both RSR and RSCC is that they are strongly correlated with metrics of model precision (*e.g*. the atomic *B* factor; see Figs. 4 ▶, 5 ▶ and 7 ▶). This means that it is not possible to say that high values in the RSR and (1 − RSCC) plots of 1f83 correlate with the known inaccuracies in this structure while at the same time explaining away similar high values in the plots for 3g94 and 2w96. Hence, these metrics are not optimal to validate model accuracy.

### 4.6. Caveat

Note that I am NOT saying that RSR and RSCC measure *only* precision: my point is that they are correlated with *both* accuracy *and* precision. This means that you do not know how much of the observed effect on RSR or RSCC to ascribe to lack of accuracy and how much to ascribe to lack of precision. It is instructive to consider why RSR and RSCC are correlated with both accuracy and precision.

RSR is straightforward: assuming that the standard uncertainty in the difference density σ(Δ*ρ*) is the same for all grid points, RSR can be written as
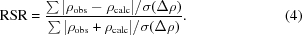
Here, the normalized difference density in the numerator is related to the log-likelihood, which is a direct measure of accuracy (see §[Sec sec5]5). On the other hand, the normalized density sum in the denominator is directly related to the model precision (see §[Sec sec6.1]6.1). Hence, RSR is correlated with both accuracy and precision.

RSCC is more complicated: again assuming the constancy of σ(Δ*ρ*) and defining
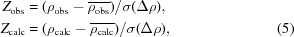
where the overbar indicates the sample mean for the ‘sample’ RSCC or the population mean for the ‘population’ RSCC, which can therefore be written as 
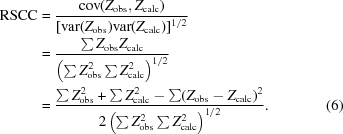
Again, the sum of squares of differences term 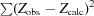 here is strongly correlated with accuracy, whereas the other terms 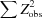 and 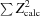 are correlated with precision. Hence, RSCC also correlates with both accuracy and precision.

## 5. The difference Fourier map and validation

The difference Fourier map has been used from the early days for small-molecule refinements and at one time it was also used routinely for macromolecular refinement: the positions and heights of difference map peaks were used to calculate shifts in atomic parameters (Watenpaugh *et al.*, 1971 ▶; Blundell & Johnson, 1976 ▶, §14.4). Even if it was not used in the refinement itself, the difference map has historically always been used to check for errors after model building or refinement, so it appears a rather obvious step to propose a validation metric based on the difference density. Indeed, it seems odd that alternative electron-density validation statistics such as RSR and RSCC have been put forward when a widely known and perfectly good (and, as I hope to demonstrate, superior) method had already existed for many years. The challenge (which turns out to be nontrivial) is to formulate an effective metric for the difference density.

As the accuracy of the model improves during model building and refinement, the difference density is systematically reduced towards a zero (or at least an insignificant) value. Hence, the *Z* score, *i.e.* the normalized difference density Δρ/σ(Δρ), being directly related to the log-likelihood, is a measure only of model accuracy, not model precision, so the use of the difference map for validation of model accuracy is an obvious step.

Note that even if the alternative RSR or RSCC metrics are used, it is still necessary to check for unexplained density (both negative and positive) in the difference map that is not in proximity to the current model, since these metrics only provide statistics for the parts of the map that are covered by the atomic model (which for a typical solvent content may be only half of the total unit-cell volume).

### 5.1. The observed distribution of the difference density

**Figure 8:**
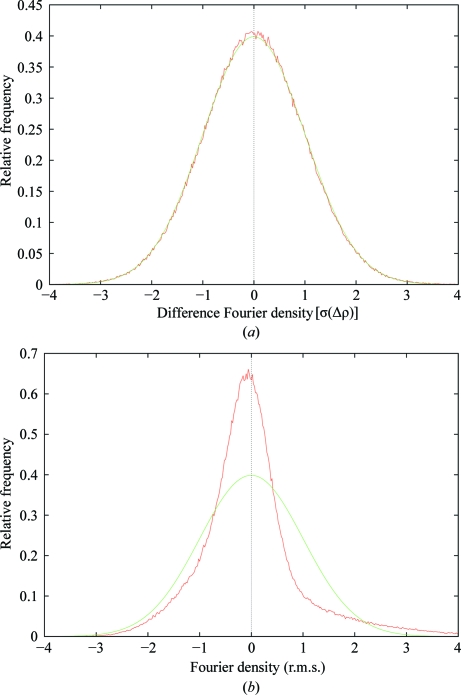
(*a*) Histogram of the 1f83 normalized difference Fourier map (red points), with the theoretical normal distribution (green curve) showing that the distribution of Δρ is very close to normal; (*b*) histogram of the 1f83 normalized Fourier map (red points), with the theoretical normal distribution (green curve) showing that the distribution of ρ_obs_ is far from normal (histograms for 3g94 and 2w96 show the same effect in each case).

A histogram (Fig. 8 ▶ *a*, red points) of Δ*ρ* demonstrates that its spatial distribution is very close to the standard theoretical normal distribution (Fig. 8 ▶ *a*, green curve). Since Δρ mostly has an expectation of zero at the completion of refinement, so that it consists mostly of random error, its error distribution is essentially the same as its spatial distribution. Note that *ρ*_obs_ obviously does *not* have a zero expectation: its expectation varies spatially in a nonrandom way, hence it does not have a normal *spatial* distribution (Fig. 8 ▶ *b*); however, it is still not unreasonable to assume that it has a normal *error* distribution (although it is unclear what value should be used for its standard uncertainty).

### 5.2. The *Q*–*Q* difference plot

**Figure 9:**
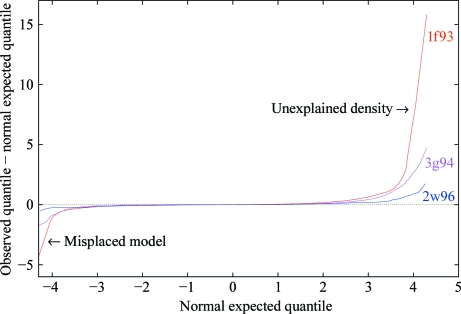
*Q*–*Q* difference plots corresponding to the histogram of Δρ in Fig. 8 ▶(*a*) for the 1f83, 3g94 and 2w96 difference Fourier maps showing deviations from the theoretical plot for a normal distribution (*y* = 0 for all *x*) in the ‘tails’ of the distribution.

A *Q*–*Q* (quantile–quantile) difference plot of the Δ*ρ* map (Fig. 9 ▶) shows deviations from normality (‘outliers’) much more clearly than the histogram plot (deviations in the ‘tails’ are greatly amplified relative to those in the central portion). A *Q*–*Q* plot (Wilk & Gnanadesikan, 1968 ▶) plots expected (*x*) against observed (*y*) quantiles (*i.e. Z* scores): if the quantile distributions differ it will show as a deviation from the straight line *y* = *x*. A *Q*–*Q* difference plot is simply a *Q*–*Q* plot with (*y* − *x*) as the ordinate (*i.e.* in place of *y*), so that an observed normal distribution plotted against a theoretical normal distribution will give the straight line *y* = 0 parallel to the *x* axis instead of the diagonal line *y* = *x*; this makes it easier to measure the deviations from normality from the plot. To construct a *Q*–*Q* difference plot, the normal expected quantile 〈*Z*〉 is plotted against the difference between the observed quantile *Z* and 〈*Z*〉, *i.e.* *x* axis = 〈*Z*〉, *y* axis = *Z* − 〈*Z*〉, where for the *i*th sample point of *n* ordered in monotonically increasing values of *Z* (equations 7 and 8; Makkonen, 2008 ▶)
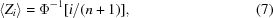


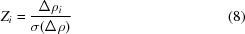
and Φ^−1^ is the inverse cumulative normal distribution function.

For a perfect normal distribution, the *Z* score is everywhere equal to its expected value, so the differences along the *y* axis = *Z* − 〈*Z*〉 are zero for all values on the *x* axis = 〈*Z*〉. Deviations from *y* = 0 indicate departures from normality. Note that this does *not* mean that the difference density is zero everywhere, rather that the observed density conforms to that expected for a normal distribution of errors. All grid points are plotted, not just those covered by the model; this means that the *Q*–*Q* plot is still a global – *not* a local – measure, since in the absence of an atomic model there is no means of identifying specific points in the plot with errors in the model.

#### 5.2.1. The *Q*–*Q* difference plot as a validation metric

We can obtain a metric of overall model accuracy in terms of consistency of the model with the difference density by simply taking the range of the vertical axis of the *Q*–*Q* difference plot, which shows the departures from normality (*i.e.* the ideal range is zero; see Table 1 ▶). The negative end of the range is a measure of misplaced atoms and the positive end of the range is a measure of unexplained density. The very large positive value for 1f83 (15.8σ) is actually owing to a single misplaced Zn atom, but even if this problem is fixed (as it is in 3g94) the large value obtained still indicates significant unexplained density, *i.e.* 4.8 standard deviations in *excess* of that expected for normally distributed random errors (usually taken as ±3σ). The *y* coordinate of the plot depends only on the deviation of the distribution of the difference density from the normal distribution; it does not depend on the solvent content or the unit-cell volume.

### 5.3. Difference density *Z* score measures local model accuracy

Model *accuracy* measures the consistency of the model with the data and the optimal measure of consistency of the model with the data is the *likelihood* of the model given the data. The optimal model is therefore the one that corresponds to the global maximum of the likelihood function, assuming that the parameterization of the model is optimal (assuming minimal overfitting to errors in the data). The likelihood is directly related to the difference density *Z* score (9) [since we are assuming a normal error distribution, the contribution to the likelihood is the Gaussian probability density function of *Z*_Δρ_, *i.e.* exp(−*Z* ^2^_Δ*ρ*_/2), omitting the arbitrary constant],
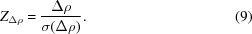
Hence, *Z* is an obvious measure of local model accuracy. Importantly, this metric is uncorrelated with model precision: imprecise local regions of the model do not necessarily show significant difference density.

#### 5.3.1. Estimation of the standard uncertainty in Δ*ρ*

The difference Fourier density Δ*ρ* is a function of three experimental variables (see §[Sec sec5.7]5.7): the observed structure amplitude *F*_o_ and the calculated amplitude *F*_c_ and phase ϕ_c_. Hence, Δ*ρ* consists of contributions from three distinct sources: (i) random experimental errors in the observations *F*_o_ (photon counting and instrumental errors, errors owing to inadequate treatment of mosaic spread and diffuse scattering, and other errors in the integration-profile model); (ii) errors in the structure-factor model itself (*i.e.* the algebraic form of the structure factor used to model anisotropy, anharmonicity, disorder and multipole effects in the atom distribution functions and scattering factors, which can only be adequately parameterized when sufficiently high-resolution data are available); and (iii) errors in the parameters of the structure-factor model (including errors in the scaling, bulk-solvent and atomic parameters and errors arising from misplaced and missing atoms and failure to adequately model disorder). Errors in the structure-factor model give rise to errors in both *F*_c_ and ϕ_c_.

The fundamental assumption in the calculation of Δ*ρ* as a true representation of the errors in the model is that *F*_o_ equals the true value of the amplitude and ϕ_c_ is the true value of the phase; it is assumed that only the amplitude *F*_c_ may differ from its true value. Hence, errors in *F*_o_ and ϕ_c_ will propagate as errors in Δ*ρ* that are not correlated with the model and therefore appear as random ‘background noise’, whereas errors in *F*_c_ are correlated with the model and therefore constitute the ‘signal’ that we wish to detect. For macromolecular structures at typical resolutions the model-error component in (*F*_c_, ϕ_c_) dominates (it is typically ∼4 times the data error; *e.g.* it explains why the precision in the data may be better than 5% but the *R* factor remains at 15–20%, even with optimal parameterization and with all the errors in the model corrected). The phase-error component of the model error contributes equally to all grid points independent of position in the unit cell (Blow & Crick, 1959 ▶; Blundell & Johnson, 1976 ▶, §12.2), with the exception of those grid points on special positions, where the error variance is multiplied by the point-symmetry multiplicity of the special position.

In practice the ‘signal’ and ‘noise’ components of Δ*ρ* can never be completely separated, particularly where the signal is comparable to or weaker than the noise. Most of the difference density arising from errors in the amplitude *F*_c_ appears in the ordered regions of the crystal since any ‘signal’ in the bulk-solvent region arising from errors in *F*_c_ from the structure-factor model will be averaged out by the solvent disorder. Consequently, the best estimate of σ(Δ*ρ*) arising from the data and phase errors should be from the bulk-solvent region.

The *CCP*4 program *EXTENDS* (Winn *et al.*, 2011 ▶) uses the method of iterative outlier rejection to determine an overall average σ(Δ*ρ*), with the overall r.m.s.d.(Δ*ρ*) as an initial estimate. An improved estimate of σ(Δ*ρ*) can then be obtained from a *Q*–*Q* plot of the density points in the bulk-solvent region: only the central portion of the plot is used (in practice points lying between ±1.5σ are used, although the precise cutoff used is not critical) in order to exclude as far as possible nonrandom difference density owing to errors in the atomic model. The gradient of the best-fit line passing through these points gives the correction factor for σ(Δ*ρ*); that is, if σ(Δ*ρ*) is already correctly estimated the gradient of the central portion of the *Q*–*Q* plot will be exactly 1 (Wilk & Gnanadesikan, 1968 ▶). In practice, this correction is found to be very small [<1.5% change in σ(Δ*ρ*) for the three cases investigated] and this has a negligible effect on the results.

### 5.4. A real-space difference density *Z* score based on the maximum deviation of Δ*ρ*

A simple and obvious method of using the difference density *Z* score as a real-space density validation metric is to take the maximum (*i.e.* peak) value over grid points within a pre-calculated limiting radius centred on each atom in a residue or split between main-chain and side-chain atoms, exactly as is performed for RSR and RSCC,
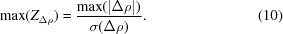
Overlaps between neighbouring atom densities are handled by partitioning the ρ_obs_ values in proportion to ρ_calc_ obtained from the truncated Fourier transform (2) of the scattering factors. The range of max(*Z*_Δρ_) is 0 (‘good’) to ∞ (‘bad’).

#### 5.4.1. Issues with the max(*Z*_Δρ_) metric: the ‘multiple comparisons’ problem

Unfortunately, the max(*Z*_Δρ_) metric as it stands is unsatisfactory as a density-validation metric for two reasons: firstly, significant statistical bias giving an overestimate of significance is inherent in taking the maximum (or minimum) value of a set of random variables, assumed here to be independent and identically distributed (iid), since the larger the sample, the higher the probability is that large deviations may occur purely through chance fluctuations. This problem of ‘multiple comparisons’ is a well established one in randomized clinical trials (Smith *et al.*, 1987 ▶), where it is possible to observe an apparently significant yet meaningless treatment effect when different tests are run comparing the treatment under trial with the best existing treatment simply by running enough tests. In the present application ‘multiple comparisons’ refers to the comparison of the set of ρ_obs_ values with their corresponding ρ_calc_ values (or equivalently comparison of the set of Δρ values with zero).

The second reason is that even after allowance for the ‘multiple comparisons’ effect on the maximum value of |Δ*ρ*|, use of the maximum value alone may also underestimate the significance because it does not take account of the possibility that there may be multiple, but an *a priori* unknown number of, grid points with significant *Z* scores in the sample. The ‘multiple comparisons’ problem has been the subject of numerous articles in the statistical literature (see Hsu, 1996 ▶, for a relatively recent and comprehensive review of the theory and methods). No single solution to the problem is appropriate in all situations simply because, as always, the answer depends on the precise question being asked of the data; hence, the method of solution must be closely tailored to the problem.

#### 5.4.2. Significance testing of the max(Z_Δρ_) metric after correction for the ’multiple comparisons’ effect

The issue of the overestimate of significance arising from the ‘multiple comparisons’ effect, when it is assumed that the variates are iid but that only one value is significant, can be addressed by application of the Dunn–Šidák correction (Sokal & Rohlf, 1995 ▶) to the maximum value. Assuming a null hypothesis of purely random errors with iid normal variates, the cumulative distribution function (CDF) of the maximum value (also known as the ‘maximum order statistic’) gives the probability that the maximum value is less than or equal to some specified value (say *x*_max_). This is obtained by noting that for this to be true each value in the sample must be less than or equal to *x*_max_ and since the distributions of the values are assumed to be independent, the required probability is that for the simultaneous occurrence of multiple independent events and is obtained by the multiplying the individual probabilities.

We are concerned here with ‘two-tailed significance tests’, in other words whether the *Z* score exceeds some threshold either in the negative or the positive direction (or equivalently whether the absolute score |*Z*| or the positive or absolute negative score taken separately exceeds some positive threshold). The cumulative probability *p* for the absolute value of the random variable |*X_i_*| is then given by the CDF for the half-normal distribution (‘two-tailed probability’),
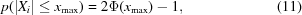
where
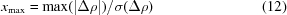
and
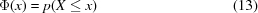
is the CDF for the normal distribution (‘one-tailed probability’, where *x* may take any value, negative or positive).

Hence, if the sample size is *n*, then since by definition all absolute values |*X*_1_|, |*X*_2_|, …, |*X_n_*| must be less than or equal to the absolute maximum value |*X*_(*n*)_|, the required CDF of the absolute maximum value |*X*_(*n*)_| is (14), *i.e.* the Dunn–Šidák corrected probability,
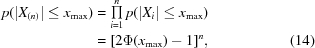
where (11) has been substituted to obtain the second expression.

As an example, suppose we observe a maximum deviation of *x*_max_ = 4σ (either negative or positive) in a sample of 100 independent values. What is the true significance of this result? From statistical tables (see, for example, http://itl.nist.gov/div898/handbook/eda/section3/eda3671.htm) *p*(*X* ≤ 4) = Φ(4) = 0.99997; hence, *p*(|*X*| ≤ 4) = (2 × 0.99997 − 1) = 0.99994 [or the standard ‘*p*-value’ = *p*(|*X*| > 4) = 1 − 0.99994 = 0.00006]. Hence, *p*[|*X*_(100)_| ≤ 4] = 0.99994^100^ ≃ 0.994 (*p*-value = 0.006). Generally, non-statisticians seem to prefer *Z* scores to *p*-values for expressing levels of significance (*e.g.* ‘*Z* = 3σ’ rather than ‘*p* = 0.0027’) and so for those people the significance of this result can probably be more easily assessed by converting it back to the equivalent normal *Z* score: for the two-tailed probability of 0.994 obtained above, the equivalent one-tailed probability is (1 + 0.994)/2 = 0.997, which corresponds (using the aforementioned table in reverse) to *Z* = 2.75σ. Hence, the apparently significant maximum value of 4σ is in reality not significant even at the usual 3σ level of significance; focusing only on the maximum value inevitably overstates the significance of the results.

#### 5.4.3. Statistically independent difference density values from resampling

A sample-size correction of the difference density score (14), as well as those versions of the score to be described in the following sections, is necessary because electron-density maps are always oversampled to avoid missing significant peaks; this means that adjacent values will be correlated and hence the assumption of independence made above would be invalid if the oversampled density values were used directly. The Shannon–Nyquist sampling theorem (Shannon, 1949 ▶) implies that the density values become statistically independent when the sampling interval is *d*_min_/2. For example, if the map is sampled at the usual interval of about *d*_min_/4 in each direction, the sample size for independence must be reduced by a factor of two in each direction, *i.e.* by about eight overall to yield the sample size *n* used in (14) and in the following sections. However, the values cannot simply be resampled on the three-dimensional grid without loss of accuracy; instead, the necessary correction can be performed very simply by resampling the ordered list of values (*e.g.* by keeping approximately every eighth value), with simple linear interpolation where the resampled value would fall in between measured values, and there will be little loss of accuracy provided that the extreme values (*i.e.* the possible outliers) are kept.

### 5.5. Real-space *Z*_Δρ_ score based on χ^2^ for all density points in the sample

The obvious alternative to using only the maximum value is to assume that all the sample values may be significant and to include all of them in the calculation of the probability. The joint probability density function (JPDF) of the absolute sample values (again assumed to be half-normal and iid) is given by 
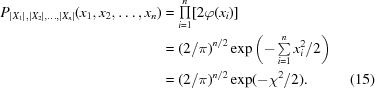
Here ϕ(·) is the usual probability density function (PDF) for the standardized normal distribution; hence, 2ϕ(·) is that for the half-normal distribution. The CDF of *χ* ^2^ for *n* degrees of freedom (*i.e.* the sample size after resampling and interpolation as described in the preceding section) is a standard textbook function: the lower regularized gamma function
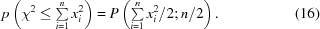
This obviously must reduce to the normal probability (11) for the specific case *n* = 1, so (16) is merely a generalization of (11) for *n* points. Notice that *P* in (16) without subscripts is the standard notation for the lower regularized gamma function and is a CDF; it should not be confused with the same symbol *P* that is conventionally used in (15) for a specific PDF or JPDF: no ambiguity arises because the latter will always be subscripted with the appropriate random variables to make it specific for the probability density function in question.

For example, suppose *n* = 100 and that |*x_i_*| = 1.1 for all *i* (in fact it is only necessary to assume that the r.m.s. value of the *x_i_* is 1.1 since this will give the same value of χ^2^). Then, χ^2^ = 100 × 1.1^2^ = 121 and *P*(121/2; 100/2) = 0.925 (*p*-value = 0.075; see http://itl.nist.gov/div898/handbook/eda/section3/eda3674.htm, using the table of upper critical values), which corresponds to a two-tailed normal *Z* score of 1.8σ and so is not significant (*i.e.* most likely just owing to random error). Now assume the same *n* but all |*x_i_*| = 1.4, so now χ^2^ = 196 and *P*(196/2; 100/2) = 0.999999967 (*p*-value = 3.3 × 10^−8^), which corresponds to a normal *Z* score of 5.5σ and so is now highly significant (*i.e.* highly unlikely to be random error).

Note that expressing the result as a normal *Z* score does not imply that the distribution is normal (in this example it is obviously a χ^2^ distribution); it is merely a more convenient way of expressing the result than using cumulative probabilities or *p*-values since most crystallographers seem to be more comfortable with *Z* scores.

The example above demonstrates that it is not necessary that any individual difference density *Z* score exceeds 3σ for the result to be significant; having all |*x_i_*| = 1.4σ is easily sufficient for it to be unlikely to be a result of random error and therefore for the score to be highly significant. This underlines the importance of taking into account all the potentially significant individual values.

#### 5.5.1. Real-space *Z*_Δρ_ score in the general case of multiple significant map values

In the case that only a few values in the sample are significant, summing the squares of all *n* deviates is likely to result in any significant signal that is present becoming diluted by the noise and so potentially being missed. This is clearly an issue with the current implementations of RSR and RSCC. For example, suppose now that *x*_max_ = 6σ with *n* = 100; also assume that the r.m.s. of the other 99 values of *x_i_* is 1. Application of the Dunn–Šidák correction to the maximum value gives a corrected *Z* score of 5.2σ and so is still highly significant. However, χ^2^ = 6^2^ + 99 × 1^2^ = 135, which for 100 degrees of freedom gives a cumulative probability *P*(135/2; 100/2) = 0.989 (*p*-value = 0.011) corresponding to a normal *Z* score of 2.5σ, which is clearly not significant according to this metric, so if we had used this method we would have missed an obvious significant error.

Clearly, everything hinges on the assumed null hypothesis, since this is the starting point for any calculation of statistical significance for which quite different estimates are likely to be obtained depending on the assumptions made. Hence, it is apparent that no single null hypothesis is capable of covering all possibilities, so it seems reasonable to propose the use of multiple null hypotheses. The main mistake that we wish to avoid is making ‘type II’ (false negative) errors, in which a false null hypothesis of no statistical significance is accepted as true (Neyman & Pearson, 1933 ▶), thus failing to spot significant errors in the model, while at the same time minimizing the frequency of ‘type I’ errors (false alarms). Therefore, we must distinguish between the possible hypotheses by selecting the one that maximizes the probability of obtaining a result less extreme than the one actually observed (*i.e.* the cumulative probability) on the assumption that the corresponding null hypothesis is true, or equivalently the one that minimizes the probability of obtaining a result more extreme than that observed (*i.e.* the *p*-value).

To this end, we take a subset of the highest values of the original *n*, say *x*_(*i*)_ for *i* = *k* to *n*, where the notation *x*_(*i*)_ indicates the value of the *i*th-order statistic (so the first method described above corresponds to the special case of the maximum order statistic for which *k* = *n*). Then, for each value of *k* = 1 to *n* we compute *χ_k_* ^2^ and its associated cumulative probability and choose that value of *k* which gives the highest probability *p*_max_ as the most likely,
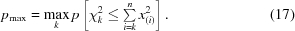
The cumulative probability of *χ* ^2^ for the case where a subset of the highest values is chosen is no longer the regularized gamma function because of the bias inherent in selecting the highest values (this is the multiple comparisons problem again). The JPDF of the order statistics of the half-normal distribution for sample size *n* is (Gibbons & Chakraborti, 2003 ▶, chapter 2)
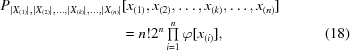
where the *n*! term comes from the number of permutations of *n* objects. The corresponding marginal CDF of χ*_k_* ^2^ is obtained in the usual way from (18), *i.e.* by integrating out all the variables *x*_(*i*)_, 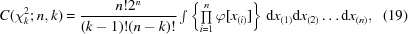
where the domain of integration is such that *x*_(*i*)_ ≤ *x*_(*k*)_ for *i* = 1 to *k* − 1, *x*_(*i*)_ > *x*_(*k*)_ for *i* = *k* + 1 to *n* and the domain of *χ_k_* ^2^ is
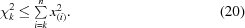
The additional factorial terms appearing in the denominator of (19) account for the fact that the orderings within the subsets of the (*k* − 1) *x*_(*i*)_ values for *i* < *k* and the (*n* − *k*) values for *i* > *k* are irrelevant; the only thing that matters is whether any value *x*_(*i*)_ is < or > *x*_(*k*)_.

Analytical integration of (19) is straightforward with respect to the variables *x*_(*i*)_ for *i* = 1 to *k*, since these are not involved in the χ*_k_* ^2^ constraint (20) and we already know the answers for the special cases *k* = 1 and *k* = *n*; however, in the general case it would appear that further progress requires numerical integration. Given that the dimensionality (*n* − *k*) of the remaining integral could be several hundred, the only feasible method available for dealing with the general case is Monte Carlo integration (*i.e.* by random sampling of the integrand; other non-stochastic methods are suitable only for dimensions less than about 20). A problem then is that the range of cumulative probabilities taken as significant falls in the very narrow range 0.9973 (corresponding to 3σ) to 1 (≡ ∞σ), so that an accuracy much better than 0.27% is required in the numerical integration; unfortunately, high accuracy is very difficult to achieve with stochastic methods when the dimensionality is high.

#### 5.5.2. Practical solution to the approximation of the real-space *Z*_diff_ score in the general case of multiple significant map values

Given the difficulty in evaluating the cumulative probability of χ*_k_* ^2^ in the general case, the following reasonable approximation (21) for the maximal value of the cumulative probability of *χ_k_* ^2^ is suggested for practical usage,
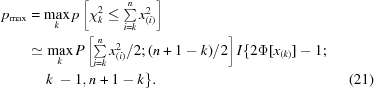
In (21) the first function *P* on the right-hand side is the lower regularized gamma function representing the usual cumulative probability of χ*_k_* ^2^ for the values *x*_(*i*)_ for *i* ≥ *k*. The second function *I* is the ‘multiple comparisons’ correction; *I* is the cumulative probability of an order statistic, namely the regularized incomplete beta function (or ‘incomplete beta integral’: Gibbons & Chakraborti, 2003 ▶, chapter 2). In the special case *k* = 1 no correction is necessary and this term is taken as 1; in the case *k* = *n* the expression reduces to the previous Dunn–Šidák expression for the maximum value (14), so (21) generalizes and gives identical results in the two previous special cases (14) and (16). In all cases the resulting cumulative probability is converted to a normal *Z* score as previously described.

Table 2 ▶ shows, for independent sample sizes *n* = 20, 100, 200 and 500, the number of independent normalized difference density values |Δρ/σ(Δρ)| at or above a specified threshold that are required to produce a significant (>3σ) RSZD score using (21), assuming that all of the other values are ±1σ. For example, for an independent sample size of 100 at least three independent values of |Δρ/σ(Δρ)| ≥ 3σ must be present for RSZD to score at least 3σ; in other words, such a distribution of values is unlikely to occur as a result of chance random errors. Note that this is after resampling, so all the counts must be multiplied by eight to obtain the corresponding actual numbers of grid points in a map sampled with spacing *d*_min_/4. Obviously, for higher density values fewer are needed to produce a significant score. Also note that the fraction of values needed at or above a given threshold value is not constant as might be expected, but depends on the sample size *n*: small samples are statistically less reliable so require a higher proportion of significant data points to achieve the same overall level of significance. Large samples require relatively fewer data points but they must have higher values to overcome the ‘multiple comparisons’ effect, where large values are more likely to occur occur purely as a result of random error.

**Figure 10:**
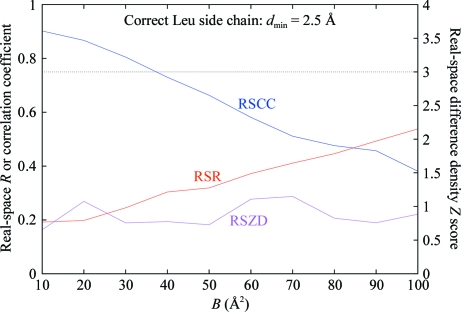
Plot of the real-space difference density *Z* score (as defined in §5.8 for a Leu side chain with simulated normally distributed random errors in the electron density (resolution cutoff *d*_min_ = 2.5 Å) based on the atomic radius limits defined in §[Sec sec5.6]5.6 as a function of the atomic *B* factor. The suggested level of significance for RSZD (3σ) is also shown (dotted line). This shows that RSZD is always well below the level of significance for a correct model regardless of *B* factor and is uncorrelated with the *B* factor. The plots of the real-space *R* and the real-space sample correlation coefficient from Fig. 7 ▶ are shown for comparison.

Fig. 10 ▶ shows the RSR, RSCC and RSZD scores plotted together as a function of *B* factor for a Leu side chain at 2.5 Å resolution, where purely normally distributed random errors in the electron density have been simulated. It is seen that the RSR and RSCC scores are both strongly correlated with the *B* factor, whereas RSZD is not; furthermore, the RSZD score falls well below the criterion for significance (3σ) independent of the *B* factor (for purely random errors the expected value of RSZD is approximately 1σ). In contrast, for RSR and RSCC no sensible criterion for significance which is independent of *B* factor can be specified.

### 5.6. The limiting radius of the atomic density

**Figure 11:**
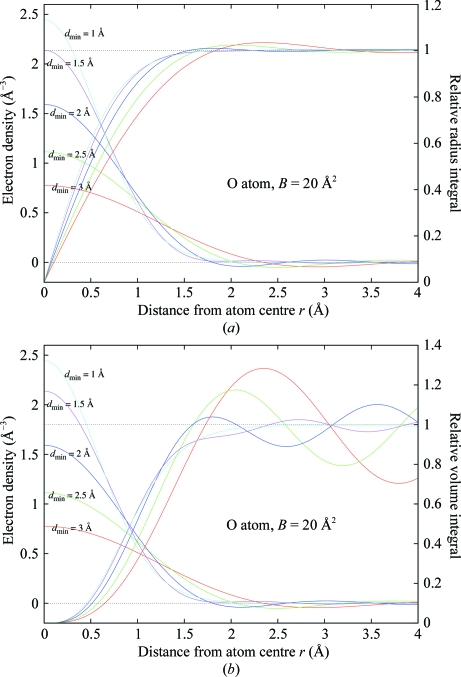
(*a*) Theoretical electron-density function and its relative radius integral plotted for an O atom (*B* = 20 Å^2^), showing the dependence on the resolution cutoff *d*_min_ (corresponding colours are used for the integral plots); (*b*) the same for the relative volume integral.

The radius enclosing the atomic density is made a function of both *B* and *d*_min_ by use of the radius integral of ρ_calc_ (22) (Fig. 11 ▶ *a*) computed by a truncated Fourier transform (2)

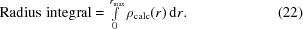



**Figure 12:**
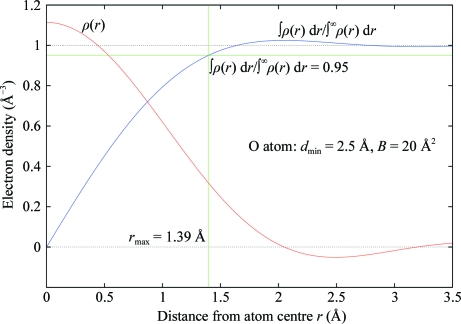
Illustration of the method used to obtain the radius limit from the radius integral; theoretical density is for an O atom (*B* = 20 Å^2^) at 2.5 Å resolution cutoff.

The radius *r*_max_ is such that the corresponding value of the radius integral is 95% of the theoretical value at infinite radius (Fig. 12 ▶).

The volume integral (23) (Fig. 11 ▶ *b*) would be the theoretically correct one to use, but unfortunately it fails to converge for large values of the radius,
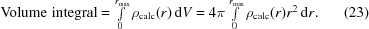



#### 5.6.1. Limiting atomic radius *r*_max_ as a function of *d*_min_ and *B* for an O atom

Table 3 ▶ shows the limiting atomic radius *r*_max_ used by various software, and that obtained using the radius integral, as a function of *d*_min_ and *B* for an O atom.

### 5.7. Difference density Fourier coefficient

If we use the ‘minimally biased’ Fourier coefficient for ρ_obs_ and the already correctly scaled *DF*_c_ coefficient for ρ_calc_ we obtain the correct Fourier coefficient for Δρ without the need for an additional scaling step, which as previously indicated if not performed correctly is very likely to introduce errors into the calculation of the density-validation metric.

For acentric reflections,
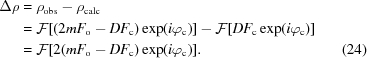



For centric reflections,
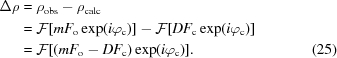
Note that using *F*_c_ in place of *DF*_c_ in the calculation of ρ_calc_ gives the wrong answer for Δρ for both acentric and centric reflections! The extra factor of 2 for acentrics relative to centrics in the Fourier coefficient of Δρ is the bias correction, *i.e.* peaks in a noncentrosymmetric difference Fourier appear at roughly half height, whereas those in a centrosymmetric map appear at full height (Blundell & Johnson, 1976 ▶, §14.2). Some refinement programs (*e.g.* *REFMAC* and *BUSTER*) use a form of the magnitude of the centric Fourier coefficient for ρ_obs_ that differs from the literature value *mF*_o_ derived theor­etically (Main, 1979 ▶; Read, 1986 ▶); the resulting ‘centric error effect’ is sufficiently large that it is detectable in a *Q*–*Q* difference plot if the space-group symmetry is sufficiently high.

### 5.8. RSZD− and RSZD+ scores

**Figure 13:**
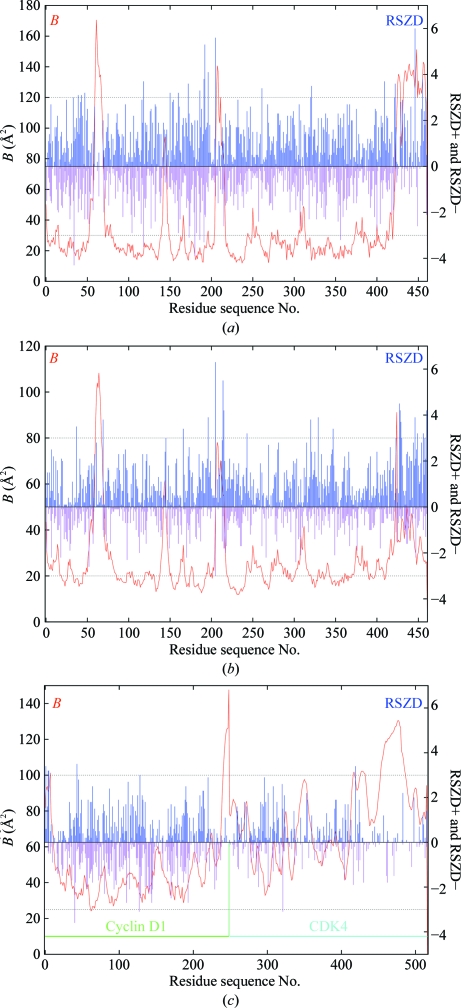
(*a*) Plot of average *B* factor and real-space difference density *Z* scores RSZD− and RSZD+ (as defined in §5.8) as a function of residue sequence number for the main-chain atoms (including C^β^) of the 1f83 structure; (*b*) the same for 3g94; (*c*) the same for 2w96. The suggested levels of significance (±3σ) are also shown (dotted lines). This shows the much higher frequency of RSZD+ scores above the level of significance for 1f83 and 3g94 compared with 2w96, indicating significant regions of positive density in the difference Fourier corresponding to errors in the models.

We can make the RSZD score a little more useful by scoring the negative and positive values of Δ*ρ* separately: ‘RSZD−’ for points with Δρ < 0 (misplaced atoms) and ‘RSZD+’ for points with Δρ > 0 (unexplained density or missing atoms). Fig. 13 ▶ shows RSZD− and RSZD+ plots for the main-chain atoms (including C^β^) of 1f83, 3g94 and 2w96. Suggested cutoff lines at ±3σ are shown; the difference in the number of outliers in the case of 1f83 and 3g94 compared with 2w96 is apparent. Table 4 ▶ shows the number and percentage of residues for each structure with RSZD− or RSZD+ scores exceeding 1σ, 2σ and 3σ thresholds. The low accuracy of the 1f83 structure compared with that of 3g94 (which itself clearly still has some issues) and 2w96 is apparent from the much higher percentage of residues with scores above each of the thresholds.

## 6. Model precision and reliability

Model precision measures the reliability of the model: if we collected a new data set and obtained from it another consistent but significantly different model, the more precise model should be the more reliable one. Various atomic and overall parameters, namely atomic number (scattering factor), site-occupancy factor and other measures of disorder, *B* factor, outer resolution limit, data precision [mean *I*/σ(*I*)] and data completeness, are all strongly correlated with model precision (Tickle *et al.*, 1998 ▶; Parisini *et al.*, 1999 ▶).

### 6.1. Validating model precision

A very simple metric of model precision that takes all correlated effects into account is the signal-to-noise ratio of the average ρ_obs_ in a specified region (26), since weak ρ_obs_ density for whatever reason clearly implies that the model is imprecise and therefore unreliable,
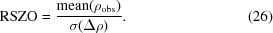
Here, the uncertainty in ρ_obs_ is assumed to be equal to σ(Δρ), not r.m.s.d.(ρ_obs_), since the latter is not a measure of the uncertainty in ρ_obs_ (it is essentially a measure of the solvent content of the crystal).

**Figure 14:**
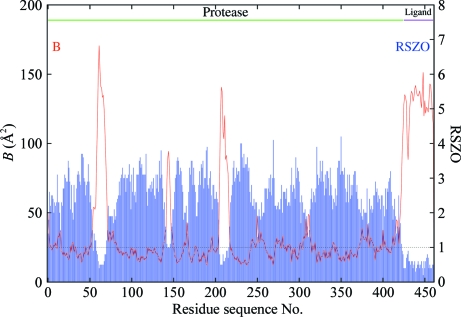
Plot of average *B* factor and RSZO score as a function of residue sequence number for the main-chain atoms (including C^β^) of the 1f83 structure. The suggested level of significance for RSZO (1σ) is also shown (dotted line). Residues with scores below the level of significance have weak average ρ_obs_ density and so should not be considered reliable.

RSZO does not correlate with model accuracy since plainly it does not depend on the model *via* ρ_calc_. The range of RSZO is 0 (‘bad’) to ∞ (‘good’). Fig. 14 ▶ shows the mean *B* factor and RSZO plot for 1f83, highlighting the regions of low precision (a suggested cutoff line at 1σ is shown). The point is that it does not necessarily follow that the regions of high *B* factor are in error, although it is true that errors are more likely in these regions.

## 7. Summary

If the goal is to validate model accuracy use a metric that is correlated only with accuracy, whereas if the goal is to validate model precision use a metric that is correlated only with precision. All RSZD (±) metrics are correlated only with accuracy; RSZO is correlated only with precision; RSR and RSCC (including variants) are correlated with both accuracy and precision. Either way, calculate your chosen validation metric accurately!

A computer program *EDSTATS* (Perl script and precompiled Linux/Intel executable with Fortran 90 source code and documentation) which computes the average *B* factor, RSR, RSCC, RSZD(±) and RSZO scores as a function of residue sequence number for a user-supplied PDB file, difference Fourier and Fourier maps (CCP4 format) may be obtained at no charge on request from the author.

## Figures and Tables

**Figure 7:**
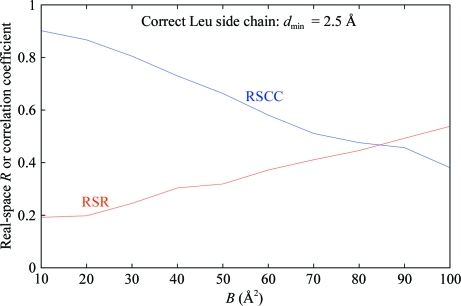
Plot of the real-space *R* and real-space sample correlation coefficient for a Leu side chain with simulated normally distributed random errors in the electron density (resolution cutoff *d*_min_ = 2.5 Å) based on the atomic radius limits defined in §[Sec sec5.6]5.6 as a function of the atomic *B* factor. This shows the correlation of RSR and RSCC with *B* factor, even for a correct model.

**Table 1:** *Q*–*Q* difference plot ranges

PDB entry	Range of vertical axis	
1f83	−4.3	15.8
3g94	−1.7	4.8
2w96	−0.5	1.8

**Table 2:** Minimum number of independent normalized difference density values |Δρ/σ(Δρ)| at or above the specified threshold *t* in a sample of size *n* that is required for the resulting RSZD score (21) to be significant (>3σ), assuming all other density values are ±1σ

	*t*							
*n*	1.5	2.0	2.5	3.0	3.5	4.0	4.5	≥5.0
20	17	5	3	2	2	1	1	1
100	25	11	6	3	2	2	1	1
200	34	14	8	4	3	2	1	1
500	49	21	12	6	3	2	2	1

**Table 3:** Radius limit *r*_max_ (Å) for an O atom as a function of resolution cutoff *d*_min_ and *B* factor by various methods

		*B* (Å^2^)								
*d*_min_ (Å)	Method	10	20	30	40	50	60	70	80	90
All	*MAPMAN*†	1.50	1.50	1.50	1.50	1.50	1.50	1.50	1.50	1.50
All	*SFALL*‡	2.35	2.67	2.95	3.21	3.45	3.67	3.88	4.08	4.27
3.5	Equation (22)	1.72	1.78	1.83	1.89	1.95	2.02	2.08	2.15	2.22
3.0		1.51	1.58	1.65	1.72	1.80	1.88	1.97	2.06	2.14
2.5		1.31	1.39	1.49	1.59	1.70	1.80	1.91	2.02	2.12
2.0		1.12	1.24	1.38	1.52	1.66	1.79	1.91	2.02	2.13
1.5		0.96	1.16	1.35	1.52	1.66	1.79	1.91	2.02	2.13
1.0		0.91	1.16	1.35	1.52	1.66	1.79	1.91	2.02	2.13

† The distributed version of *MAPMAN* uses *r*_max_ = 1.5 Å (independent of element, *d*_min_ and *B*). The Uppsala Electron Density Server (http://eds.bmc.uu.se/eds) version of *MAPMAN* uses variable *r*_max_ (Professor G. Kleywegt, personal communication).

‡ *SFALL* uses *r*_max_ = 2.5(*B* + 25)^1/2^/2π Å (independent of element and *d*_min_).

**Table 4:** Number and percentage of protein residues with RSZD− and RSZD+ scores exceeding 1σ, 2σ and 3σ thresholds for 1f83, 3g94 and 2w96 (excluding heteroatoms)

	RSZD−			RSZD+		
PDB entry	>1σ	>2σ	>3σ	>1σ	>2σ	>3σ
1f83	276 (59.9)	92 (20.0)	19 (4.1)	346 (75.1)	194 (42.1)	74 (16.1)
3g94	162 (35.1)	30 (6.5)	5 (1.1)	264 (57.3)	103 (22.3)	39 (8.5)
2w96	186 (36.0)	58 (11.2)	9 (1.7)	174 (33.7)	45 (8.7)	11 (2.1)
